# 

**DOI:** 10.1192/bjb.2019.75

**Published:** 2020-04

**Authors:** Linda Gask

**Affiliations:** Emerita Professor of Primary Care Psychiatry, University of Manchester, UK. Email: linda.gask@manchester.ac.uk

One of the most difficult things about writing a book for general readers on mental health (and, more particularly, psychiatry) is knowing how to structure it – and where to begin. The authors of this very comprehensive book, a liaison psychiatrist (Ellen) and a writer–comedian (Deveny), have both experienced depression and they share their tales with considerable frankness and humility. However, instead of beginning with these engaging stories, they choose to start with a section on diagnosis and classification – which might unfortunately deter some from continuing.

There are really useful sections on how to talk to friends who you think might need help and what happens when you go to see a mental health professional. However, my particular favourite has to be ‘clues your shrink is a dud’, which warns against those who claim excessive certainty, have a guru mentality and are excessively expensive. And therein lie clues that this book doesn't originate in the UK, but hails instead from Australia. The text has clearly been edited for the UK edition, with reference to clinical commissioning groups, mental health trusts and a list of UK organisations from which further help can be sought. However, there is, for example, no reference to the problems faced by those from UK ethnic minorities, the section on drugs mentions neither ‘skunk’ or ‘legal highs’ and the classification system is, of course, DSM.

Readers of this book would learn a great deal about mental health and illness from a biopsychosocial perspective. They might, however, be left with an idea that there is considerably more choice of professional and therapist in the National Health Service than in reality – although this may of course be true if they can pay. Personally, I don't see any problem in asking your GP if they are good at mental health, and I wish it was easier to ask for second opinions. The authors tell us ‘remember you are in charge!’ but for many people seeking help it rarely feels that way.

